# Reprioritising global mental health: psychoses in sub-Saharan Africa

**DOI:** 10.1186/s13033-023-00574-x

**Published:** 2023-03-28

**Authors:** O. O Omigbodun, G. K Ryan, B Fasoranti, D Chibanda, R Esliker, A Sefasi, R Kakuma, T Shakespeare, J Eaton

**Affiliations:** 1 Department of Psychiatry and Centre for Child and Adolescent Mental Health, College of Medicine, University of Ibadan, Ibadan, 200212 Oyo State Nigeria; 2 Department of Population Health, London School of Hygiene and Tropical Medicine, Centre for Global Mental Health, Keppel Street, London, WC1E 7HT UK; 3 Research Support Centre, Faculty of Medicine and Health Sciences, University of Zimbabwe, Avondale, Harare, Zimbabwe; 4 Mental Health Department, University of Makeni, Lunsar-Makeni Highway, Makeni, Sierra Leone; 5 Department of Mental Health, Kamuzu University of Health Sciences, P/Bag 360, Blantyre, Malawi; 6 Department of Population Health, London School of Hygiene and Tropical Medicine, International Centre for Evidence in Disability, Keppel Street, London, WC1E 7HT UK; 7 CBM Global Disability Inclusion, Dr.-Werner-Freyberg-Straβe 7, 69514 Laudenbach, Germany

**Keywords:** Sub-Saharan Africa, Psychosis, Global Mental Health, Human Rights

## Abstract

Arthur Kleinman’s 2009 *Lancet* commentary described global mental health as a “moral failure of humanity”, asserting that priorities should be based not on the epidemiological and utilitarian economic arguments that tend to favour common mental health conditions like mild to moderate depression and anxiety, but rather on the human rights of those in the most vulnerable situations and the suffering that they experience. Yet more than a decade later, people with severe mental health conditions like psychoses are still being left behind. Here, we add to Kleinman’s appeal a critical review of the literature on psychoses in sub-Saharan Africa, highlighting contradictions between local evidence and global narratives surrounding the burden of disease, the outcomes of schizophrenia, and the economic costs of mental health conditions. We identify numerous instances where the lack of regionally representative data and other methodological shortcomings undermine the conclusions of international research carried out to inform decision-making. Our findings point to the need not only for more research on psychoses in sub-Saharan Africa, but also for more representation and leadership in the conduct of research and in international priority-setting more broadly—especially by people with lived experience from diverse backgrounds. This paper aims to encourage debate about how this chronically under-resourced field, as part of wider conversations in global mental health, can be reprioritised.

## Introduction

**Table:** 

**Box 1. Excerpt from “Global mental health: a failure of humanity"**	
*Ground zero in global mental health is not the 15% [sic] of the global burden of disease accounted for by the cost of mental disorders… Globalised cultural changes have brought about important reductions in the discrimination, fear, and isolation surrounding depression and anxiety disorders in many countries…. [yet] conditions for people with psychosis, dementia, and mental disability remain horrendous most everywhere. (Kleinman 2009, p. 603)*	

In 2009 Arthur Kleinman [1] published a commentary criticising “the moral failure of humanity” that has allowed people with severe mental health conditions to live under some of the worst possible conditions in all countries of the world and throughout history (p. 604). Kleinman argued that “ground zero” in global mental health should not be the epidemiological or economic arguments that tend to favour common mental health conditions such as mild to moderate depression and anxiety [2], but instead urged for action to protect the basic rights of those in the most vulnerable situations.

More than a decade later, depression remains the most commonly studied mental health condition in global mental health and an “implicit priority” of the field (Misra et al. 2019, p.1) [3]. Depression appears in more than twice as many empirical studies on global mental health (29.7%) compared to psychoses (12.6%) [3]. High-profile efforts in global mental health in recent years have explicitly focused on depression; for example, the 2016 World Bank-World Health Organization (WHO) event “Out of the Shadows: Making Mental Health a Global Priority”, as well as the Wellcome Trust’s 2019 announcement of a £200 million Mental Health Priority Area (though this has since been expanded to include psychosis as well as depression and anxiety) [4–6]. The editors of *Lancet Psychiatry* (2020) have observed that even prior to the Coronavirus outbreak, “offering desperately needed help to those experiencing severe mental illness was too often secondary to the more prominent discourse around easily scaled and delivered talking therapies for common mental disorders” (pp. 463) [7].

We do not wish to criticise action on depression—a condition with which the authors have substantial personal and professional experience and agree is deserving of attention (not least of all because symptoms of depression and psychosis so frequently co-occur) [8]. However, it does appear that people with severe mental health conditions like schizophrenia and bipolar disorder are at risk of being left behind in global mental health and in international development more broadly [9–12]. Epidemiological and economic data should not be the sole basis for priority-setting, which must also take into account arguments around human rights and social justice [1]. But it certainly does not help the case for psychoses that those data are often based on controversial, outdated studies [13, 14] and blunt models [15] that may not reflect the present-day realities of mental health in sub-Saharan Africa [16].

Sara Cooper (2014) has argued that in our enthusiasm for promoting evidence-based medicine, following a hierarchy of evidence that privileges larger-scale and more resource-intensive quantitative methodologies over more localised and often qualitative study designs, we may be neglecting other approaches to thinking about mental healthcare in sub-Saharan Africa [17, 18]. In this paper, we attempt to highlight some of the contradictions between local evidence and global narratives that privilege common mental health conditions, pointing out the omissions and methodological weaknesses of large-scale research on the Global Burden of Disease, the epidemiology of schizophrenia, and the economic costs of mental health conditions. In the process, we draw on research and experience from sub-Saharan Africa to make the case for more attention to psychoses in this region. We focus mainly on severe mental health conditions like schizophrenia, schizoaffective disorder and similar primary psychotic disorders, as well as bipolar disorder, which is frequently accompanied by psychosis [19].

## Methods

We carried out a narrative review of literature on psychosis in low and middle income countries (LMICs) as part of the initial scoping and development of a new Health Research Programme Consortium (RPC), SUCCEED Africa (Support, Comprehensive Care and Empowerment of People with Psychosocial Disabilities in sub-Saharan Africa) between 2018 and 2020 [20]. In the process, we identified several landmark studies whose conclusions appeared to contradict evidence and experience from the region, as observed by SUCCEED’s local Principal Investigators and managers (for example during a 2019 RPC Theory of Change workshop) and other scholars of mental health in sub-Saharan Africa (e.g., Oye Gureje [16], Jonathan Burns [14]). This critical review is the result of subsequent efforts to investigate these contradictions and make recommendations for further research, by an international, multidisciplinary group of SUCCEED researchers with either professional or lived experience of psychosis in sub-Saharan Africa.

### Key concepts, terminology and scope

The term “psychosis” is a phenomenological concept operationalised by various diagnostic classification systems to describe an individual’s experience of symptoms (e.g., delusions, hallucinations, disorganised thinking) that characterise a number of “psychotic disorders” [21], but may also occur in individuals with other mental and neurological disorders (e.g., depressive and anxiety disorders, bipolar type I and II [19, 22, 23]), or even in the absence of any diagnosable mental disorder (e.g., as a result of sleep deprivation, certain physical health conditions like HIV/AIDS, malaria and typhoid, some medications like chloroquine and corticosteroids, alcohol and illicit drug use, etc. [24–26]). The plural “psychoses” is often used as a catch-all referring to some or all of these varied categories, further blurring the lines between different states of being, symptoms and diagnoses.

On the one hand, this ambiguity may allow for more inclusive discussions of psychosis in the mental health literature, acknowledging concerns around “labelling” with a particular (or indeed any) mental disorder as well as long-standing debates surrounding the validity and reliability of psychiatric diagnoses, both of which are magnified when taking a cross-cultural perspective [27, 28]. On the other hand, trying to represent profoundly diverse experiences under the heading of “psychosis” or “psychoses” may have a homogenizing and ultimately reductionist effect. Even our attempt to limit this review to specific diagnostic categories is undermined by ongoing concerns regarding the clinical and biological heterogeneity of conditions like schizophrenia and bipolar disorders, reified by recent findings in genomics and neuroimaging [29–33]. These issues are further compounded when attempting to speak about an entire class of disorders. For example, the International Classification of Disease’s “schizophrenia and psychotic disorders” groups together conditions as diverse as “acute and transient psychotic disorder” (in which episodes typically last from as little as a few days to one month) and “continuous schizophrenia” (in which symptoms are present for a minimum of one year, with very little reprieve) [21]. Painting these conditions with the same brush obscures crucial differences in risk factors, treatment and care, outcomes and measurement [34], in the experiences, needs and priorities of those affected, and ultimately in the barriers they face in making their voices heard—with important implications for mental health research, advocacy, programming and policy.

Although cognisant of these limitations, we concentrated our review of “psychoses” in sub-Saharan Africa on primary psychotic disorders and bipolar disorder for pragmatic reasons. WHO groups together psychosis and bipolar disorder under the “psychoses” module of its mental health Gap Action Programme (mhGAP) Intervention Guide [35]. This ambiguity originally served a functional purpose, allowing for non-specialists to identify and treat psychotic symptoms following a common algorithm, without necessitating a formal diagnosis [36]. However, mhGAP also has a complicated “social life” that extends beyond its clinical application [18]. Increasingly, this group of psychoses is used to frame broader discourses in global mental health, sometimes under the heading of “severe mental disorders” (a category which also includes moderate to severe depression by WHO’s definition [37], though in practice is often used interchangeably with “psychoses”). As outlined above, it is our aim in this paper to question the implicit prioritisation of common over severe mental health conditions by unpicking the evidence that is selected for “global” studies and further knowledge translation by international bodies like WHO; hence, we focus on the diagnoses that tend to feature most prominently in these.

On a related note, as members of a consortium that co-produces mental health research in sub-Saharan Africa, we wish to preface this critical review by expressing our discomfort with the overuse of deficit-based language in the mental health literature [38]. We generally feel obligated to replicate this terminology in order to accurately represent the research under discussion. Where possible without substantially altering the original meaning of the text, we adopt person-first language that emphasises individuals’ lived experience (which may refer to past or present experience) and use the more general terms “mental health conditions” and “psychosocial disabilities”, as opposed to “disorders” or similar. This is in keeping with guidance that two of the authors (GR, JE) have produced for international development organisations [9]. However, we recognise that these alternatives may not be accepted by all readers, or even by all members of our consortium (for example, several authors question the distinction of certain conditions as “severe” by WHO). Terminology remains an ongoing discussion within SUCCEED, which includes team members from different cultural traditions and professional backgrounds across five countries. We are still in the process of developing our own consensus-based style guide for research communications.

## Results

### Global burden of disease: can we trust the DALY?

The Global Burden of Disease (GBD) metric (“Disability-Adjusted Life Years”, or “DALYs”) combines disability (“Years Lived with Disability”, or “YLDs”) and mortality data (“Years of Life Lost”, or “YLLs”) to rank health conditions in terms of their “disease burden” at a population level [39]. The 1990 GBD study that attributed more than 10% of DALYs to psychiatric conditions [40] is often credited with catalysing the development of global mental health as a field [41–44]. In particular, the inclusion of unipolar depression among the top five greatest contributors to the global disease burden shocked the international development community and continues to feature heavily in advocacy and communications about global mental health.

Yet the use of GBD metrics to define priorities in global mental health is increasingly under criticism. First, advocates have argued on semantic grounds that the language of “burden” implies that people with mental health conditions are problems that need fixing [42]. Second, methodologists have questioned the data sources and modelling techniques employed to calculate the GBD. Since the 1990s, critics like Richard Cooper and colleagues have argued that in the absence of sufficient data from sub-Saharan Africa, the GBD numbers are “guesstimates… constrained largely by the need to avoid conflict with previous estimates” (1998, pp. 208) [45]. Of regional GBD estimates published in 1997, Cooper et al. complain that mortality data was based on vital registrations from South Africa alone, representing just 1.1% of the population of sub-Saharan Africa [45–47]. While advancements in health and demographic surveillance systems have helped to improve mortality estimates over the past two decades, they cannot substitute for adequate civil registration and vital statistics system [45–48]. As of 2003, only five countries in sub-Saharan Africa were able to report “useable” mortality data from their vital registrations to the WHO [48, 49].

The lack of regionally-representative data continues to call into question the validity of GBD results for mental health, specifically. Brhlikova, Pollock and Manners (2011) report that the national estimates used in 2000 to calculate the GBD for depression came from just 40 of 191 WHO member countries. While studies from 15 of 52 European countries (28.85%) were included in this dataset, there were only three studies from 46 African countries (6.52%), and each of these three covered a single village or town, as opposed to a nationally representative sample [50]. Examining the data sources for schizophrenia in the 2019 GBD study [51], there are five studies from three countries in sub-Saharan Africa: Botswana, Ethiopia and Zanzibar (United Republic of Tanzania) (Table 1). Four of these studies are more than twenty years old, two come from the same district in Ethiopia, and none uses a nationally representative sample.

**Table 1:** Sources of data on schizophrenia as causes of death and illness in sub-Saharan Africa, Global Burden of Disease Study, 2019

Author (year)	Country	Sample
Awas et al. (1999) [52]	Ethiopia	501 community members from Butajira district (predominantly rural)
Fekadu et al. (2015) [53]	Ethiopia	359 people with schizophrenia from Butajira district (predominantly rural)
Kebede et al. (1999) [54]	Ethiopia	1,420 people with a suspected mental health condition from one administrative division of Addis Ababa (urban)
Bondestam et al. (1990) [55]	Zanzibar (United Republic of Tanzania)	10,766 community members from Unguja (mixed rural and urban) and Pemba (predominantly rural) islands
Ben-Tovim et al. (1986) [56]	Botswana	2,526 community members from six villages in the Chobe region (rural)

### Prevalence and incidence

Why does the lack of timely, regionally-representative epidemiological data matter for psychoses, specifically? There is a common misconception that rates of psychoses are fairly consistent between countries, perhaps obviating the need for further epidemiological research, but this is not the case. Global meta-analyses estimate < 1% lifetime prevalence of psychotic disorders, but reviewers repeatedly highlight the heterogeneity of this data [57–59]. Hairong He and colleagues’ (2020) analysis of the changing GBD of schizophrenia from 1990–2017 found the greatest rise (> 130%) in both incident cases and DALYs was in sub-Saharan Africa (specifically, Central and Western Africa) and was only partially attributable to population growth [60]. However, they again caution that data from the least-developed countries tend to have the greatest data limitations.

Indeed, this is the central premise behind the research of the INTREPID consortium [61, 62]: without more research from LMICs, we cannot claim to know the most basic facts about the global epidemiology of psychoses, rendering the calculation of more sophisticated measures (such as the DALY) highly suspect. INTREPID has recently published results of epidemiological studies comparing rates of untreated psychotic disorders at study sites in Nigeria (Ibadan), India (Kancheepuram) and northern Trinidad [62]. Overall, age- and sex-standardised rates were approximately three times higher in northern Trinidad compared to the other two sites. However, participants from the Nigerian and Indian sites were more likely to meet diagnostic criteria for schizophrenia (51% Nigeria, 47% India, 39% Trinidad), while brief and affective psychoses were much more common in northern Trinidad. The authors conclude that research on psychoses should not be generalised from high-income countries (HICs) to LMICs, though it’s worth noting that there were statistically significant differences in rates of psychoses between the two LMIC sites as well.

### Morbidity and mortality

While an examination of prevalence data helps to illustrate critiques regarding the representativeness of GBD estimates, further consideration of co-morbidities and mortality data highlights the limitations of the GBD studies’ approach to modelling. According to evidence from mainly HICs, people with severe mental health conditions have 10–20 years shorter average lifespan compared to the general population [15, 63], and this gap may be worsening [64]. In Southern Ethiopia, people with schizophrenia or bipolar disorder die approximately 30 years younger than the general population, mainly from infectious diseases [53]. A recent analysis of World Health Survey data shows there is a statistically significant difference (*p* < 0.0001) in the prevalence of multi-morbidities (two or more physical health conditions) between people with diagnosed psychotic conditions (36.0%), subclinical psychosis (21.8%) and general population controls (11.4%) in LMICs specifically [65, 66]. Around the world, people with severe mental health conditions are more likely to experience physical health conditions, they often receive a lower standard of health care for these conditions, and they have more difficulty adhering to treatment, resulting in poorer health outcomes [63, 67]. Unsanitary conditions and abusive practices in institutions [68, 69], as well as polypharmacy [70, 71] and inadequate management of the sometimes dangerous side effects of anti-psychotic medications and mood stabilisers [37, 67], also present serious health risks. Meanwhile, people with severe mental health conditions are also at greater risk of suicide and are more likely to be victims of violence [63, 67, 72–74]. For example, among women with schizophrenia attending an outpatient clinic in Southern Nigeria, 75% had experienced intimate partner violence [66, 75]. Yet calculations of YLLs do not account for all of the 14.3% of deaths worldwide that may be attributable to mental health conditions [76].

Daniel Vigo and colleagues (2016) have shown that when the attribution of mortality to severe mental health conditions and other methodological limitations are addressed, the disease burden for mental health conditions (13.03% DALYs) is roughly on a par with that of cardiovascular and circulatory disease (13.5% DALYs), currently the number one contributor to total GBD [15]. The team responsible for the calculation of GBD estimates for mental and substance use disorders, Whiteford, Ferrari and Vos (2016), agree that the standard of attributing deaths solely to their direct cause is a limitation [77]. For example, they have stated that there is not yet sufficient data “to the standard required for inclusion in the GBD” (pp. 403) to identify what proportion of non-lethal self-harm to attribute to mental disorders [77]. In a separate analysis by Ferrari et al. (2014), schizophrenia had the third highest risk of suicide (pooled relative risk of 12.6%), exceeded only by major depression (19.9%) and cocaine dependence (16.9%) [78]. After accounting for suicide, schizophrenia moved up four places in the 2010 GBD rankings, from the 43^rd^ biggest cause of disease burden to the 39^th^. However, the authors acknowledge that there were no data on the distribution of suicides attributable to mental and substance use disorders in sub-Saharan Africa, again limiting the generalizability of their results. This is undoubtedly due in no small part to the widespread stigmatization of suicide in the region, and particularly the criminalization of suicide in several countries [79].

### The WHO schizophrenia studies: are outcomes really better in sub-Saharan Africa?

Over nearly three decades, the WHO carried out several large international studies of schizophrenia, starting with the International Pilot Study of Schizophrenia launched in 1967 [80], and later followed by the “Ten Country Study” [81] and the International Study of Schizophrenia [82, 83]. The legacy of the WHO schizophrenia studies continues to shape how we think about psychoses as a public health issue in LMICs. One enduring message is that outcomes for people with schizophrenia in LMICs seem to be better than in HICs, fuelling arguments that efforts to improve mental health care for psychoses in LMICs are at best futile, and at worst arrogant and imperialistic—perhaps even damaging. Yet the WHO studies have been critiqued on many fronts, and a 2012 meta-analysis found that after excluding these studies, the difference in clinical and social recovery outcomes in low- and lower-middle income countries, compared to upper-middle income countries and HICs, was no longer statistically significant (*p* = 0.632) [84]. One of the most glaring issues with the WHO studies—and with the more recent 36-country Worldwide Schizophrenia Outpatient Health Outcomes (W-SOHO) study that claims to support the WHO studies’ findings [85] is lack of representation from sub-Saharan Africa. In the first two WHO studies, Ibadan, Nigeria was the only African site; in the last WHO study and the W-SOHO study, there were no African sites [14, 86]. As Jonathan Burns highlights, the rapidly changing social, political and economic landscapes in African countries and subsequent changes to risk and protective factors render these decades-old studies in Nigeria obsolete [14]. There were also other methodological shortcomings, mostly rooted in the heterogeneity of psychosis discussed above, which may have resulted in the over-representation of participants with acute conditions with better outcomes. In addition to inconsistencies between sites, including diagnostic differences and the potential for selection bias, these surveys did not account for attrition bias [13, 14]. Yet attrition can be high in studies of people with schizophrenia [13, 87], due to a wide range of factors: impairments related to the condition itself; social barriers, such as stigma; structural barriers, such as high rates of homelessness and incarceration; and poor physical health outcomes, including premature mortality [88].

In a 2008 review of the literature on schizophrenia outcomes in LMICs, Alex Cohen and colleagues argue that the picture is “far more complex” than the WHO studies might suggest (pp. 229) [13]. This review identified four African studies in addition to the WHO study in Ibadan. In Butajira, Ethiopia, 10.3% of a schizophrenia cohort with an 84.4% follow-up rate died over the 1–4 year follow-up period [89–91]. In Ilesa, Nigeria, 7.8% died in a study with an 81.0% follow-up rate over 2.1–3.2 years [92]. Though mortality rates were not reported, follow-up rates were similar for a thirteen-year retrospective study conducted in Abeokuta, Nigeria (85.7%) [93], as well as a prospective study with a two-year follow-up in Cape Town, South Africa (84.2%) [94]. Meanwhile, the Ibadan, Nigeria site included in the WHO studies had only a 69.0% follow-up rate over two years, and mortality was not reported [81]. As Cohen and colleagues argue, premature mortality is surely among the worst possible outcomes of schizophrenia, and it is unwise to draw any conclusions from studies with high and unexplained attrition rates. Further, their review highlights the dangers of generalizing the WHO study results not only across LMICs generally or sub-Saharan Africa specifically, but even within countries, as follow-up rates varied greatly between the WHO study in Ibadan and the two other studies with Nigerian samples.

### Care for people with psychoses in sub-Saharan Africa

The controversial assumption that outcomes of psychoses might be better in LMICs than HICs is sometimes credited to the care available for people with psychoses in these countries—whether from the formal mental health system, traditional or religious healers, or families and communities. While recognising that each of these resources can play a crucial and often very supportive role, we would caution against overly romanticised views of the care currently available.

### Medical care

Although not a panacea, clinical interventions do exist for the management of psychoses and can be delivered in LMIC settings [2]. However, access to treatment is often limited, as are treatment options [66]. In a given year, only 31% of people with schizophrenia in LMICs receive treatment; in low-income countries, it is just 11% [95]. As Laura Asher (2018) notes in a review of recent evidence on schizophrenia in LMICs, qualitative studies from Ethiopia [96] and Tanzania [97] reported erratic supply chains and difficulties paying for medication as substantial barriers to engagement with formal mental health care [66]. In the case of long-acting injectable antipsychotics, which many consider to be more convenient and discrete than oral medications [91, 98], there is speculation that commercial interests may be interfering with global supplies (see, for example, the US pharmaceutical company Lannett’s 1,650% increase in the price of fluphenazine [99]).

A population-based study in rural Ethiopia found that more than 90% of those identified with schizophrenia or bipolar disorder had never received treatment [100]. Even after integrating mental health care into primary care in a nearby area, less than a third (29.8%) of people with psychoses who accessed these services received minimally adequate treatment, defined by the programme as at least one prescription at a “therapeutic level” plus four or more follow-up appointments [101]. Many African countries rely heavily on first-generation anti-psychotic medications with few alternatives available for those who experience distressing side effects, so even this definition of “minimally adequate treatment” (derived from previous studies in HICs [100]) is perhaps over-generous. Critics of the prevailing biomedical paradigm in mental health would also take issue with any implication that medication alone is adequate for people with psychoses.

### Traditional healing

Local healing traditions (e.g., traditional or spiritual healing) are ubiquitous in many LMIC settings, and often the first port of call for help-seeking. Evidence from sub-Saharan Africa indicates that approximately half of people seeking mental health care first visit a traditional or spiritual healer [102]. However, even these alternatives can be inaccessible to many. For example, the Nigerian Survey of Mental Health and Well-being found only 8% of people with “seriously disabling disorders” had received any form of clinical treatment or alternative care over the past 12 months [103]. Further, it is important to note recent findings of a meta-analysis suggesting that the solutions offered by healers have less efficacy for psychoses than for common mental disorders [104]. Consequently, there is a tendency for people with psychoses to be subjected to more drastic and sometimes abusive practices, such as shackling and physical assault, over much longer periods of time [68]. In the Ilesa study described above, more than half of recorded deaths took place at traditional healers’ compounds [13, 92]. Burns (2012) also cites his own previous studies from KwaZulu-Natal, in which those who attributed first-episode psychosis to supernatural causes or consulted a traditional healer before presenting to formal mental health services had more negative symptoms and spent longer without formal treatment [14, 105, 106]. On the other hand, recent studies of collaboration between healers and formal health care providers have shown promising results for people with psychoses in Ghana [107] and Nigeria [108].

### Informal care from families and communities

In the absence of adequate services, including social welfare, much of the caring responsibility for people with psychoses falls on families and particularly on women and girls [66]. The purported difference in outcomes between LMICs and HICs is often attributed to greater social acceptance, the tolerance of the extended family, and the quality of human relationships, especially in rural areas [13]. Yet from her work in rural Ghana, anthropologist Ursula Read suggests that the picture is more varied [109]. Read shares examples of desperate families, fearful of extremes of behaviour such as violence and vagrancy, shackling people with severe mental health conditions in their family compounds. While she emphasises that families are typically pushed to shackling as a last resort in rural areas where few alternatives are available, she also observes instances where restraint can serve as a form of punishment. Family support has its limits, as noted by Cohen et al. (2008) in reference to a retrospective study of social outcomes of people with schizophrenia in Abeokuta, Nigeria: 4% of subjects were homeless or in unstable housing [13, 93]. The original authors Gureje and Bamidele (1999) were surprised by the finding, but concluded that prolonged illness could lead to breakdown of family support networks [93]. In Ethiopia, Senair Ghebrehiwet and colleagues (2020) have also identified important gender differences, with families offering less social support to women with schizophrenia, compared to men [110]. In a review of recent evidence on schizophrenia in LMICs, Asher (2018) highlights two small-scale but in-depth qualitative papers from South Africa that contest some key assumptions around the nature and availability of informal care for people with schizophrenia in LMICs: families did provide care, but sometimes felt obliged to do so; care was not always available due to caregiver employment, sickness or death; and caregivers found it very hard to support medication adherence, particularly given factors such as violence, substance use and difficulties ensuring food supplies [66, 111, 112].

We share these examples not to demonise families or devalue their important contributions to care, but rather to emphasise that their contributions should not be taken for granted. People with psychoses in sub-Saharan Africa deserve more and better options, as do their families.

### The economic “burden”: are we focusing on the right costs?

Compounding the limitations of the Global Burden of Disease studies with the many assumptions necessary for top-down economic modelling, in 2011 the World Economic Forum (WEF) calculated the economic “burden” of neuropsychiatric conditions based on the previous year’s DALY estimates [113]. Neuropsychiatric conditions accounted for more money lost from the global economy than any other non-communicable diseases, including cardiovascular diseases: $16.3 US trillion between 2010 and 2030, with $7.3 US trillion coming from LMICs, mainly due to losses in economic productivity. These figures have featured prominently in communications for global mental health advocacy. While they are not disaggregated by condition, they are generally interpreted as making an economic argument mainly for common mental health conditions—as explained by Vikram Patel in his article on Universal Health Coverage for schizophrenia (2016, pp.885–6):

*The best available interventions* [for schizophrenia] *are neither curative nor lifesaving, rendering them less attractive when compared with interventions such as antidepressants or antiretrovirals. It is therefore not surprising that of all the mental disorders, depression, which is associated with high burden and cost-effective interventions and for which the counter-factual case of the cost of inaction is compelling, has attracted most attention *[2].

What goes unsaid in Patel’s article is how cynicism regarding the productive potential of people with psychoses may also contribute to a less “compelling” “counter-factual”. Psychosis typically onsets in adolescence or early adulthood [114] and can evolve into a chronic, life-long condition. This means that precisely at the time when young people are preparing to enter the workforce or further their education, their professional development is interrupted [115]. One figure commonly cited in reports by UN agencies (though based on US research by the National Institute of Mental Health) is that the unemployment rate for people with severe mental health conditions is 70–90%, higher than virtually any other group of persons with disabilities [10, 116]. There is very little research on employment and mental health-related workplace discrimination in LMICs, though at least one cross-cultural study comparing the US and China claims that American employers may actually be less hesitant about hiring people with psychoses [117]. What this means for unemployment rates in sub-Saharan Africa, however, we should not speculate.

African economies are largely informal and heavily agricultural, which could feasibly offer more flexible opportunities for people with mental health conditions to contribute economically, for example by helping to cultivate a family farm. But these contributions are extremely difficult to measure and model, and are often left out of employment figures. Findings of a systematic review by Huey Yi Chong et al. (2016) suggest that in African studies (both from Nigeria [118, 119]), indirect costs such as losses to productivity by people with schizophrenia account for a much smaller percentage of the economic burden of schizophrenia than in HICs and in LMICs in other world regions (Table 2) [120]. More research is needed, from more countries, to understand whether these trends are artefacts of methodological differences or reflective of a very different economic reality for people with psychoses in sub-Saharan Africa.

**Table 2:** Differences in the ranges of direct vs. indirect costs attributed to schizophrenia across major world regions and income levels, adapted from Chong et al. (2016)

Region	Income level	Cost contribution to total cost (percentage range)		
		Direct medical cost	Direct nonmedical cost	Indirect cost
Africa	LMICs	73^a^–85%	2%^a^	12–27%
Americas	HICs	19–35%	< 0.1–14%^b^	50–81%
Asia	LMICs	18–32%	0.1–10%	71–82%
	HICs	14–28%	< 0.1–1%	72–85%
Europe	HICs	24–87%	2–12%^b^	8–76%

^a^Amoo and Ogunlesi (2005) include some direct nonmedical costs in their calculation of direct medical costs, and do not report direct nonmedical costs separately

^b^Some studies’ direct nonmedical costs reported as “not applicable”

Either way, we must be careful not to export discriminatory beliefs (and unrepresentative data) about the potential of people with lived experience of psychoses from HIC to LMIC economies. Indeed, Lisa Cosgrove and others have criticised the imposition of capitalist economic arguments altogether, expressing resentment over the “neoliberalization of mental health” that “promotes an ethics of utility rather than an ethics of care” and frames distress as “economically burdensome” (2019, n.p.) [43]. Even if we put aside these broader critiques, it is undeniable that the costs of providing inappropriate, ineffective or inadequate care for psychoses are substantial, not just to individuals, but also to health systems and families.

### Costs to health system

Inpatient psychiatric care is the most expensive mental health service, and 80% of government mental health expenditure in LMICs is spent on psychiatric hospitals [121]. The WHO estimates that for schizophrenia the cost of hospital-based mental health care is 33–55% higher when compared to a community-based service model [2, 122, 123]. People with psychoses are among those most likely to be admitted for inpatient psychiatric care—often involuntarily [124] and to be readmitted after leaving inpatient care [125, 126]. For example, at Uganda’s only psychiatric referral hospital, nearly two-thirds (62.7%) of patients are diagnosed with a psychotic disorder at first contact [127]. In Nigeria, a diagnosis of schizophrenia is a predictor of psychiatric readmission [128], and the average cost of a single psychiatric hospital admission ($3675 USD) is equivalent to the cost of 90 outpatient visits [129, 130]. Action on the deinstitutionalization and decentralization of mental health care for people with psychoses could increase coverage, lower per capita costs, and help to address some of the most egregious human rights violations that occur in institutions [131].

### Costs to families

In 43% of African countries—the largest percentage of any world region—families pay mostly or entirely out of pocket for mental health care [121]. Meanwhile, most caregivers for people with schizophrenia in sub-Saharan Africa are female and unemployed, despite many being of working age (mean age 46.3), and report that the severity and duration of the illness has a negative impact on their own employment and income [132]. A study from Ghana found that the average monthly cost of care for a person with a severe mental health condition was $160.00 USD per patient, in addition to indirect costs at $133.31 USD per month. Meanwhile, the average monthly income reported by households of people with mental health conditions was just $184.48 USD [133]. Another study carried out in Nigeria found that over half (55.8%) of families of people with schizophrenia or a major affective disorder reported that caring for their relative had a moderate to major financial impact on their households [134]. Consequently, nearly a quarter (23.2%) resorted to either selling property or taking loans. The extreme poverty faced by people with psychoses and their families can threaten their very survival. In a rural district of Ethiopia, people with schizophrenia or bipolar disorder are nearly three times more likely (odds ratio 2.8) to experience severe household food insecurity [66, 135]. Confronted with these harsh realities, it is difficult to understand why severe mental health conditions like psychoses do not feature more heavily in discussions on poverty reduction in sub-Saharan Africa.

## Discussion

### Key findings

To summarise, there are a number of reasons why psychoses in sub-Saharan Africa may have historically been deprioritised in global mental health, some of which are perhaps rooted in the constraints of international research carried out to inform “evidence-based” decision-making. We don’t really know the true prevalence or incidence of psychoses in sub-Saharan Africa, their outcomes or their costs—and what little we know about morbidity and mortality is not necessarily taken into account when calculating the “burden” of psychoses in either epidemiological or economic terms.

In 2020, Nanna Weye and colleagues from Canada and Denmark published an editorial declaring, “These [Global Burden of Disease] methods have been good for mental health—*but not good enough*” (pp. 103, italics authors’ own) [136]. The same is true of psychoses in sub-Saharan Africa. While the disability weights employed by the GBD studies do favour conditions like schizophrenia, they have not historically been based on empirical research into the lived experiences of people with psychoses around the world (though this is starting to change) [137]. The relative dearth of high-quality epidemiological studies from this region means that we cannot count on the basic prevalence and incidence data so essential for the calculation of DALYs. This issue is further compounded by rudimentary methods for the attribution of mortality, which are especially consequential for psychoses. According to a global meta-analysis, the pooled relative risk of mortality among people with psychoses (2.54) is significantly higher (*p* < 0.05) than among those with other mental health conditions, such as mood disorders (1.86) and anxiety (1.43) [76].

A further example of the detrimental effects of unrepresentative and methodologically flawed epidemiological research is the conclusion drawn from the WHO schizophrenia studies that outcomes are more favourable in LMICs than in HICs. Several researchers have questioned this, calling for more rigorous studies of the long-term course of psychoses in LMICs [138]. As researchers, clinicians, and people with lived experience in sub-Saharan Africa today, this picture of a better outcome does not reflect our experience. We take pride in the resourcefulness and dedication of those who provide care—both formal and informal—for people with psychoses in these challenging circumstances. However, we cannot condone what medical anthropologists like Paul Farmer have deemed the employment of culture as an excuse for inaction in global health [139]. This does not mean that we believe in the superiority of HIC mental health care. Rather, we agree with former WHO Director of the Department of Mental Health and Substance Abuse Shekhar Saxena [140]: “When it comes to mental health, all countries are developing countries. No country has mental health care services worked out quite satisfactorily” (Davies 2018, pp. 1509).

Finally, the weaknesses of the GBD studies are quantified in dollars and cents through top-down economic modelling that does not necessarily take into account high unemployment rates or the importance of the informal economy in sub-Saharan Africa, and might not value the ways in which people with psychoses, their families and community organisations might contribute to society—economically or otherwise. Meanwhile, we know the cost of care as it is currently provided is not affordable to either health systems or households, and that it drives people in already vulnerable situations toward catastrophic health expenditure and extreme poverty.

### Implications and recommendations

Our findings point to the need not only for more research on psychoses in sub-Saharan Africa, but also for more representation and leadership from the region in the conduct of this research and in international priority-setting more broadly. Decolonising global mental health is a vital end unto itself [141, 142], but we also trust these efforts will lead to more cautious interpretation and application of supposedly “global” evidence that all too often treats geographic disparities in mental health research as a mere methodological shortcoming. The consequences are very real, shaping the narratives that drive decision-making in a chronically under-resourced field—and ultimately the lived experiences of people with mental health conditions around the world. Their voices, especially, should be at the forefront of global mental health research, service development, training and advocacy, as argued by the Pan African Network of People with Psychosocial Disabilities in their 2011 Cape Town Declaration [143]: “There can be no mental health without our expertise. We are the knowers and yet we remain the untapped resource in mental health” (PANUSP 2014, pp. 385). Tapping this resource will require more targeted funding for inclusive psychosis research led by African researchers and more support for people with lived experience to be involved—including as leaders of their own research. These efforts must also recognise and embrace the incredible diversity that exists among those with lived experience of psychosis, acknowledging that some voices are harder to hear than others.

This is not to say that we would encourage research for research’s own sake. On the contrary, we agree with calls from African disability advocates for “no survey without service” (Schneider et al. 2002, pp. 182) [144], particularly for people with psychosocial disabilities, who are often exceptionally underserved and marginalised even within the global disability movement. Further—and in line with a social model of disability—we would argue that these services should extend beyond clinical treatment and toward ensuring full participation in society. While African countries appear to be leading the way in research on the integration of mental health into non-specialist health care via mhGAP [145, 146], critics have questioned whether mhGAP goes far enough in addressing the diverse needs of people with schizophrenia, bipolar disorder and other severe mental health conditions [147]. The good news is that there are, increasingly, promising examples of innovation upon which we can draw in this region: collaborative care spanning traditional, spiritual and allopathic medicine in Nigeria and Ghana [107, 108]; formal peer support for people with severe mental health conditions in Kenya, Tanzania and Uganda [148–151]; community-based rehabilitation for people with schizophrenia in Ethiopia [152, 153]; and many more.

Moreover, we believe that much can be achieved by improving access to opportunities that already exist in other sectors. To illustrate: an evaluation of the Malawi Incentive Programme’s conditional cash transfer scheme not only showed improvements in mental health outcomes, but that recipients with the poorest mental health had the greatest improvements (approximately four times the average effect size) [154]. Yet people with severe mental health conditions are often excluded from poverty-reduction interventions [10, 155]. Peer researchers on SUCCEED have also highlighted the importance of increasing accessibility in schools and workplaces, for example by offering more flexible study opportunities and working hours to allow for daily self-care, as well as longer interruptions when needed. The empowerment of people with psychosocial disabilities to claim their own rights is essential to identify and address these and other instances of exclusion in the region.

## Conclusions

We agree with Kleinman [1] that our starting point in global mental health should be more about the human rights of people in the most vulnerable situations, and less about the big numbers generated to guide global decision-making—not only because failure to do so perpetuates a long-standing “moral failure of humanity” (2009; pp. 604), but because the numbers themselves are deeply flawed. Over twenty years ago, Richard Cooper [45] and colleagues wrote of the early GBD studies, “If these data are wrong, the consequences are likely to be most damaging for the very populations unrepresented in the fact-gathering process” (1998; pp. 210). In this paper, we have examined the GBD and other landmarks in international mental health research that may have inadvertently undermined action on psychoses, drawing on mounting—if sometimes fragmented—evidence about psychoses in sub-Saharan Africa. In the process, we call for more research on psychoses to be focused on sub-Saharan Africa and driven by African researchers and people with lived experience, in particular. However, we also agree with Kleinman that action to promote the rights of people with psychoses is well overdue and cannot wait any longer. Research must be coupled with concrete efforts to increase access to holistic services within and beyond the heath sector, and to address the many barriers to full and equal participation in society faced by people with psychoses and other psychosocial disabilities in the region.

## Data Availability

All data generated or analysed during this study are included in this published article.
